# Using transgenic reporters to visualize bone and cartilage signaling during development *in vivo*

**DOI:** 10.3389/fendo.2012.00091

**Published:** 2012-07-18

**Authors:** Chrissy L. Hammond, Enrico Moro

**Affiliations:** ^1^Departments of Biochemistry, Physiology and Pharmacology, University of Bristol, Bristol, UK; ^2^Department of Biomedical Sciences, University of Padova, Padova, Italy

**Keywords:** bone, cartilage, reporter, technology, transgenic, zebrafish, bone, cartilage, transgenic, reporter, zebrafish

## Abstract

Green fluorescent protein was first used as a marker of protein expression *in vivo* 18 years ago, heralding the beginning of what became known as the Green Revolution. Since then, there has been an explosion in the number of transgenic lines in existence, and these transgenic tools are now being applied to skeletal research. Advances in transgenesis are also leading to increasing use of new model organisms for studying skeletogenesis. Such new models include the small teleosts zebrafish and medaka, which due to their optical translucency offer imaging possibilities in the live animals. In this review, we will introduce a number of recent advances in genetic engineering and transgenesis and the new genetic tools that are currently being developed. We will provide examples of how zebrafish and medaka transgenic lines are helping us to understand the behavior of skeletal cells *in vivo*. Finally, we will discuss future prospects for the application of transgenic technology to skeletal research.

## GENERAL INTRODUCTION

Skeletal tissues have proved particularly difficult to image at a cellular level in live animals due to the depth and mineralization of the tissues. In recent years, there have been advances in hard tissue and skeletal imaging in live organisms, for example the increased sensitivity of high resolution magnetic resonance imaging (HR-MRI; Patsch et al., 2011) and improvements to CT scanning (Chappard et al., 2011). However, while these techniques give improved detail about bone structure and micro-architecture, they do not tell us much about the behavior of cells within the skeletal tissues.

The first mouse transgenic line was created in 1974 (Jaenisch and Mintz, 1974). Since then, transgenic mice have been instrumental in increasing our understanding of the lineages in skeletal development, via the use of lacZ reporters for lineage analysis and to assess the requirement of specific genes in skeletal lineages. The further introduction of conditional gene deletion techniques has considerably facilitated the analysis of target bone and cartilage related genes. In these cases a driver line carrying a recombinase (Cre) driven by cartilage or bone-specific promoters is crossed to a genetically modified mouse strain carrying a “floxable” cassette to be targeted to the gene of interest. Through this technique for example Col2a1-Cre and Col1a1-Cre lines have been used to drive recombination in chondrocytes and osteoblasts, respectively (Terpstra et al., 2003; Zha et al., 2008). From these studies using transgenic lines we have garnered a wealth of information on the genes required for the specification and maturation of chondrocytes (e.g., Leung et al., 2011) and osteoblasts (reviewed by Long, 2012). However, despite the increasing availability of genetic tools, generating transgenic lines in mice by injection into the pronucleus, or more commonly by injection of engineered stem cells into blastocysts remains technically demanding, time consuming and relatively expensive (Miller, 2011).

Since mice develop *in utero*, following dynamic signaling events in real time during bone development is technically almost impossible. As such, studies at a cellular level typically require post mortem analysis of the skeleton by histology or immunohistochemistry. Therefore, much of what we know about the real time *in situ* dynamics of chondrocyte and osteoblast behavior, gene expression, migration, and maturation has come from *in vitro* studies. However, it is difficult to know to what extent these features mirror the environment *in vivo,* which is rich in cues from the surrounding tissues, and from the complex *in vivo* mechanical environment. Attempts to replicate this environment in culture have been made through seeding of cells onto biomimetic scaffolds (Tampieri et al., 2011). However, the development of tools to dynamically track gene expression and signaling pathway activity in live animals has remained highly desirable.

## TELEOST BONE DEVELOPMENT

Zebrafish, along with another teleost species medaka, have long been used as model organisms for developmental biology. In the early stages of skeletal research in fish, research focused primarily on lineage tracing (Schilling and Kimmel, 1994) and forward genetic screening (Driever et al., 1996; Haffter et al., 1996). More than 50 mutants with defective cartilage and skeletal development were identified from the first large-scale screens (Neuhauss et al., 1996; Piotrowski et al., 1996; Schilling et al., 1996). However, a particular advantage to using teleosts comes from the ability to image skeletal development in real time in developing larvae (or even increasingly in more mature fish), using fluorescent transgenic reporter lines.

The zebrafish craniofacial skeleton contains bones of both dermal and chondral origins, which arise from different progenitor cells, such as neural crest cells and mesodermal cells (Schilling and Kimmel, 1994). By contrast, some the vertebrae originate through the mineralization of the notochord sheath, while other bones, such as those located in fins, arise through a cartilage template (Bird and Mabee, 2003). Both key regulators of skeletal development and the control of the major signaling pathways are highly conserved between mammals and teleosts (reviewed by Apschner et al., 2011). Therefore, findings in fish are highly likely to be applicable to mammalian osteogenesis.

## ADVANCES IN TRANSGENESIS

In recent years there have been multiple advances in our ability to generate transgenic reporters in zebrafish, radically reducing the time to generate new lines. Traditionally, transgenic reporters were generated by microinjection of linearized plasmid DNA, containing the coding sequence of a reporter protein [typically green fluorescent protein (GFP)] immediately downstream of a minimal promoter fragment for the gene of interest (Higashijima et al., 1997). However, this approach suffered from a number of limitations, in particular the low efficiency of germline integration.

Advances to the technology have included the introduction of the Gateway system and the production of compatible plasmids that can be used in zebrafish (Kawakami et al., 2004; Villefranc et al., 2007), I-SceI cloning, whereby introduction of meganuclease sites increased the efficiency of germline integration (Grabher and Wittbrodt, 2008). More recently, improvements have been achieved by bacterial artificial chromosome (BAC) recombineering, in which fluorophores and Tol2 transposase sites are introduced into a BAC containing the gene or promoter of interest. The frequency of germline integration is improved by this method, while the constructs for transgenesis can be generated and injected into zebrafish embryos in less than 3 weeks (Bussmann and Schulte-Merker, 2011; Suster et al., 2011). As such, the number of available transgenic lines generated through these methods is likely to increase exponentially in the future.

Tol2 transgenesis has also been used in recent years for enhancer trapping. An Enhancer Trap construct drives a reporter, often eGFP, controlled by a minimal promoter in a vector that can be inserted into the genome at random; if the insertion occurs near to an enhancer it will produce tissue-specific expression of the reporter. Various enhancer trap screens have been documented in fish (see Table 1) and a number of these lines show specific reporter expression in skeletal tissues, for example, the line ET 33-1B (http://plover.imcb.a-star.edu.sg/webpages/ET33-1B.html), which is specifically expressed in the craniofacial skeleton (of both dermal and chondral origins). In fish and frog models, site-directed transgene integration is a more recent development; however, systems are now established for these model organisms. In frog this is accomplished via a FLP-FRT recombinase-mediated transgenesis method (Zuber et al., 2012), while in fish site-directed intramolecular transgenesis can be achieved using the Cre-Lox system (Mosimann and Zon, 2011) or by using PhiC31 integrase (Lister, 2011). The utility of the latter system is further increased by the ability to utilize the PhiC31 system for efficient recombinase-mediated cassette exchange (RCME), whereby fluorophores can be efficiently excised and replaced with other fluorophores or by Cre (Hu et al., 2011).

**Table 1:** Table of existing transgenic lines and resources relevant to skeletal development.

Structure/cell type labeled	Gene or response element	Line name	Reference
Osteoclast	*Cathepsin K*	*Tg(CTSK -DsRed)*	Chatani et al. (2011)
		*Tg(ctsk:mEGFP)*	To et al. (2012)
Osteoblast	*Sp7/osterix*	*Tg(Ola.Sp7:NLS-GFP)zf132*	Spoorendonk et al. (2008)
		*Tg(Ola.Sp7:mCherry)zf131*	DeLaurier et al. (2010)
		*Tg(sp7:EGFP)b1212*	
Osteoblast	*Bglap/osteocalcin*	*Tg(Ola.Osteocalcin.1:EGFP)hu4008*	Knopf et al. (2011)
Chondrocyte	*Col2a1a*	*Tg(Col2a1aBAC:mcherry)hu5900*	Hammond and Schulte-Merker (2009)
		*Tg(-1.7col2a1a:EGFP-CAAX)nu12*	Dale and Topczewski (2011)
Joints	*Trps1*	*Tg(trps1*^*J1271aGt*^)	Talbot et al. (2010)
Cartilage	*Col18a1*	*Tg(16Hsa.COL18A1-Mmu.Fos:EGFP)*	Kague et al. (2010)
Neural crest derivatives including	*Fli1*	*Tg(Fli1:eGFP)y1*	Lawson and Weinstein (2002)
chondrocytes and osteoblasts			
(and endothelial cells)			
Neural crest-derived skeleton	*Sox10*	*Tg(-1252sox10:GFP)ba5*	Dutton et al. (2008)
(and pigment cells)	*Sox10-Cre*	*Tg(-4725sox10:Cre)ba74*	Rodrigues et al. (2012)
Osteoclast	*TRAP*	*Tg(TRAP:GFP)*	Chatani et al. (2011)
Osteoclast	*RANKL (heat shock inducible)*	*Tg(rankl:HSE:CFP)*	To et al. (2012)
Preosteoblasts	*Cyp26b1*	*Tg(cyp26b1:YFP)hu5786*	Spoorendonk et al. (2008)
Intervertebral discs	*Twhh (shhb)*	*Tg(-5.2shhb:GFP)mb1*	Haga et al. (2009)
Intervertebral discs	*Twist*	*Tg(twist:EGFP)*	Inohaya et al. (2007)
Osteoblasts (conditional ablation line in medaka)	*Osx/Sp7*	*Tg(Osx:CFP-NTR)*	Willems et al. (2012)
Bmp responsive cells (including craniofacial elements)	BMP response element	*Tg(BRE:GFP)*	Collery and Link (2011)
		*Tg(bre:egfp)*^*pt510*^	Laux et al. (2011)
Branchial arches and notochord	*Cyp26a1*	*Tg(cyp26a1:eYFP)nju1/ +*	Hu et al. (2008)
Wnt responsive cells (including craniofacial elements)	Wnt response element	*Tg(7xTCF.XlaSiam:nlsmCherry)ia5*	Moro et al. (2012)
**Name of resource**	**Method of generation**	**Link**	**Reference**
Ztrap	Enhancer trap	http://kawakami.lab.nig.ac.jp/ztrap/	Urasaki et al. (2008),
			Kotani et al. (2006),
			Kawakami et al. (2004, 2010)
ZETRAP	Enhancer trap	Now merged with ZETRAP2.0 (website below)	Choo et al. (2006)
ZETRAP2.0	Enhancer trap	http://plover.imcb.a-star.edu.sg/webpages/geneexpression.html	Kondrychyn et al. (2011)
Enhancer TRAP	Tol2 Enhancer trap	No web resource.	Fisher et al. (2006)
Crezoo	CreERT2 insertions	http://crezoo.crt-dresden.de/crezoo/	Hans et al. (2011)

## TOOLS FOR STUDYING SKELETOGENESIS IN TELEOSTS

**FIGURE 1:**
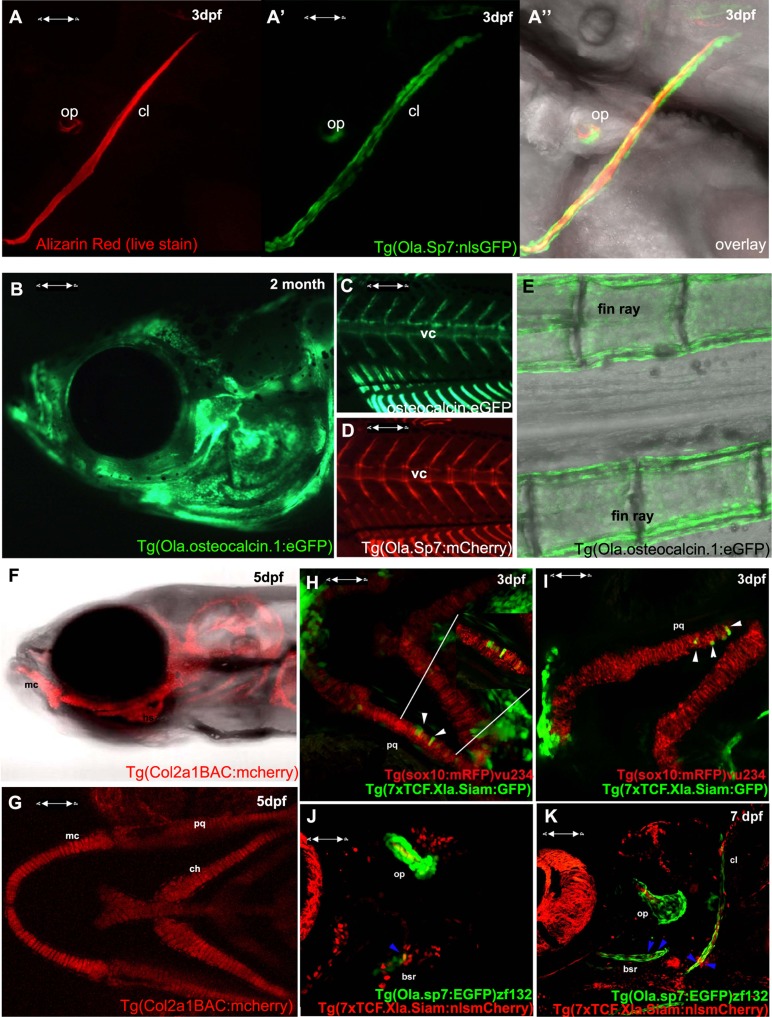
**Visualizing transgene expression in live fish during skeletogenesis**. **(A)** Alizarin red live staining in a lateral view of a zebrafish at 3 dpf, A′ Osterix/sp7 activity Tg(Ola.Sp7:nlsGFP)zf132 in the same fish. A′′ overlay showing an Alizarin red-positive mineralizing core (red) surrounded by osteoblasts (green). **(B)** Osteocalcin/bglap Tg(Ola.Osteocalcin.1:EGFP) hu4008reporter activity in the head of a 2-month-old zebrafish. **(C)** Osteocalcin Tg(Ola.Osteocalcin.1:EGFP)hu4008activity in the vertebral column of a 19-day-old zebrafish. **(D)** osterix/Sp7 Tg(Ola.Sp7:mcherry)zf131 activity in the same 19-day-old zebrafish as C. **(E)** Tg(Ola.Osteocalcin.1:EGFP)hu4008reporter expression in the caudal fin rays of a 2-month-old zebrafish. **(F,G)** Cartilage visualization in a 5-day-old zebrafish with a col2a1a reporter, Tg(Col2a1aBAC:mCherry)hu5900 **(F)** shows a lateral view of the head, **(G)** is a ventral view. **(H,I)** Representative 3 dpf double transgenic Tg(sox10:mRFP)vu234;Tg(7xTCF.Xla.Siam:GFP)ia4 showing neural crest cell-derived chondrogenic cells expressing the Wnt reporter transgene (white arrowheads). **(J,K)** Representative 4 dpf **(J)** and 7 dpf **(K)** double transgenic Tg(sp7:EGFP)b1212;Tg(7xTCF.XlaSiam:nlsmCherry)ia5 showing isolated *osterix* positive cells coexpressing the Wnt reporter transgene (blue arrowheads). All images are confocal Z-stack projections. **(H,I)** Ventral views with anterior to the left. **(J,K)** Lateral views with anterior to the left. vc, vertebral column; mc, Meckel’s cartilage; bsr, branchiostegal ray; ch, ceratohyal; cl, cleithrum; op, opercle; pq, palatoquadrate.

In terms of tools for the study of skeletogenesis, many labs have been generating an increasing number of transgenic tools to aid zebrafish and medaka skeletal research. These include a variety of transgenic reporter lines to mark skeletal lineages at different stages of differentiation; such as the chondrocyte reporters, *Tg*(*Col2a1aBAC:mCherry*)*hu5900* (Hammond and Schulte-Merker, 2009; **Figures 1F–G**) and *Tg*(1.7col2a1a:mCherry-caax; Dale and Topczewski, 2011), an increasing number of transgenic lines specific for osteoblasts, such as the *osterix*/*sp7* reporter lines *Tg*(*sp7:EGFP*)*b1212* and *Tg*(*Ola.Sp7:NLS-GFP*)*zf132* (Spoorendonk et al., 2008; DeLaurier et al., 2010; **Figures 1A,D,J,K**) and *osteocalcin*/*bglap* reporter line, *Tg*(*Ola.osteocalcin:EGFP*)*hu4008* (Knopf et al., 2011; **Figures 1B,C,E**). There are also available reporters for osteoclasts such as the *cathepsin K* reporter *Tg*(ctsk*:mEGFP*; To et al., 2012) and for joint fate, such as *trps1*^*J1271aGt*^ (Talbot et al., 2010). Live zebrafish can be incubated with dyes that bind mineralized tissue such as Alizarin red or calcein, which allows monitoring of bone matrix formation *in vivo*. Combinations of these lines, along with calcein or Alizarin red stains, allow dynamic imaging of skeletal development and cell maturation in the living fish (**Figures 1A–G**).

Recently, an emerging approach to dynamically dissect the *in vivo* activation or repression of endogenous signaling pathways is the generation of biosensor reporter fish, expressing reporter proteins (GFP, mCherry, DsRed, Kaede, YFP) under the control of minimal signaling pathway responsive elements (Dorsky et al., 2003; Parsons et al., 2009; Schwend et al., 2010; Collery and Link, 2011; Laux et al., 2011). In these transgenic lines synthetic arrays of repetitive responsive elements are fused upstream of a minimal promoter, such as Thymidine kinase or Epstein–Barr Virus terminal protein 1, and drive the expression of the reporter gene with a spatiotemporal resolution depending upon the strength of the minimal promoter and the stability of the reporter protein itself. These tools have been used both in drug screening tests and for analysis of the various signaling pathways in genetic mutants.

The BMP, Hedgehog, and Wnt signaling pathways have all been previously shown to actively control vertebrate chondrogenesis through their concerted actions (reviewed in Goldring et al., 2006). We have recently generated a novel Wnt/beta-catenin reporter fish, expressing the eGFP or mCherry protein under the control of a multimerized array of seven TCF/Lef binding sites upstream to a *Xenopus leavis* minimal *siamois* promoter, showing its application to test Wnt agonists and antagonists, as well as trace the dynamics of neural crest-derived cell migration during fish growth (Moro et al., 2012). This reporter has been also used to highlight the mechanism through which the proliferation of posterior lateral line primordium (PLLP) cells is maintained during neuromast production (Valdivia et al., 2011). By combining this transgenic line with the *Tg*(*sox10:mRFP*)*vu234* (Kirby et al., 2006) Tg(Ola.Sp7:NLS-GFP)zf132 (Spoorendonk et al., 2008), we have been able to identify clusters of neural crest-derived cartilage elements and osteoblasts expressing Wnt reporter activity (**Figures 1H–K** and data not shown).

Specific ablation of target cells in a temporarily controlled fashion can be achieved through use of the nitroreductase (NTR) system, in which the coding sequence for a gene encoding a NTR enzyme that can render prodrugs such as metronidazole (Met) cytotoxic, is expressed under the control of a promoter of interest. Usually, a fluorophore is also expressed to enable simultaneous cell tracking (Pisharath and Parsons, 2009). This system has been used to generate a medaka transgenic line whereby NTR is expressed under the control of the osteoblast *Sp7*/*osterix* promoter, in which osteoblasts can be visualized by CFP and ablated following treatment with Met. Experiments in this line have demonstrated a novel role for osteoblasts in forming the borders between vertebral centrae (Willems et al., 2012). Additionally, induction of skeletal genes can also be achieved at a desired time by use of a global heat shock inducible system; for example, in the medaka an inducible osteoclast model has been used to induce widespread osteoclastogenesis by induction of the RANKL promoter under the control of a heat shock element (*rankl*:HSE:CFP) leading to an osteoporotic phenotype (To et al., 2012). Then To et al. visualized osteoclast behavior by using second osteoclast-specific transgene, ctsk:mEGFP.

Increasingly, research using zebrafish is giving us an insight into understanding the signals that control skeletal behavior at the single cell level *in vivo*. Three examples, in which use of transgenic lines, have been key to dissecting phenotypes at a cellular level are briefly discussed below.

### THE ROLE OF *CYP26B1* IN PATTERNING THE AXIAL AND CRANIOFACIAL SKELETON

Forward genetic screens identified two mutants: *stockstief* (Spoorendonk et al., 2008), which was identified on the basis of fusions of the axial rings that generate the future vertebrae, and *dolphin*, identified by the “beak-like” shape of the jaw (Piotrowski et al., 1996). Both mutations were subsequently revealed to be lesions in the same gene, *cyp26b1*, an enzyme which degrades retinoic acid (RA; Laue et al., 2008; Spoorendonk et al., 2008). By generating a *cyp26b1BAC:yfp* construct and injecting this into embryos carrying the Sp7 reporter transgene, the authors were able to show that *cyp26b1* colocalizes with the osteoblast marker *Sp7*/*osterix* in craniofacial skeletal elements. Together these mutants demonstrated that tight control of RA levels is required for the correct positioning of osteoblasts both in craniofacial elements (Laue et al., 2008) and axial skeleton (Spoorendonk et al., 2008). In the axial skeleton use of a nuclear-localized osteocalcin transgenic line allowed both the number and localization of osteoblasts in the vertebral column to be quantified, demonstrating that the number of osteoblasts is unchanged in mutants. This suggests that the overmineralization of the vertebrae is caused by a change in osteoblast activity rather than their number (Spoorendonk et al., 2008). Moreover, it has recently been demonstrated that Cyp26 enzymes are required to control local RA metabolism during cranial suture formation in zebrafish, mice and humans (Laue et al., 2011), supporting the view that the requirement of Cyp26 enzymes for the correct activation of osteoblasts is conserved between teleosts and humans.

### BONE REGENERATION OCCURS VIA DEDIFFERENTIATION OF OSTEOBLASTS IN THE ZEBRAFISH FIN

Bone has a limited capacity for repair in mammals, and bone healing, following, e.g., a fracture or break, is believed in mammals to be achieved through activation of a resident population of osteogenic precursor cells and recapitulation of developmental ossification pathways (Ferguson et al., 1999; Dimitriou et al., 2011). Salamanders and fish have a more robust capacity for repair and regeneration of many tissues (Tanaka and Reddien, 2011), with fin regeneration in the zebrafish frequently used as a model for regenerative studies (Tal et al., 2010). However, it was unknown whether the “dedifferentiated” cells that form a wound blastema, following fin amputation, and which give rise to the regenerated structures, are multipotent or lineage restricted. Using different transgenic approaches to mosaically label cells (Tu and Johnson, 2011) or throughout the organism (Knopf et al., 2011), two groups showed that the dedifferentiated cells in the blastema only give rise to cells of the same lineage, demonstrating that, although the cells in the wound blastema can dedifferentiate and proliferate, they maintain their lineage restriction throughout this process (Knopf et al., 2011; Tu and Johnson, 2011). Very recently, a *de novo* origin of osteoblasts during fin regeneration, following genetic ablation of existing osteoblasts, has demonstrated that the cellular origin of appendage bones can be different according to tissue damage (Singh et al., 2012).

### CARTILAGE MATRIX CONTROLS TIMING OF ENDOCHONDRAL OSSIFICATION

The timing of events such as chondrocyte maturation with osteoblast differentiation and activation is critically important during endochondral ossification, during which multiple signaling pathways are activated. Cartilage matrix is rich in sulfated proteoglycans, and both heparin and chondroitin proteoglycans are known to associate with and regulate diffusion of signaling factors within the mouse cartilage growth plate (Settembre et al., 2008; Gualeni et al., 2010; and reviewed by Mackie et al., 2011). Recently, Eames et al. (2011) demonstrated that two zebrafish mutants (*xylt1* and *fam20b*) which produce lower levels of chondroitin sulfate proteoglycans, undergo premature endochondral ossification of their cartilage elements. They demonstrated, using transgenic lines and *in situ* hybridization, that both mutants showed premature perichondral osteoblast differentiation, which could be abolished by crossing them to an Indian hedgehog (*ihha*) mutant line. These findings, taken together with those from mouse models, demonstrate that cartilage matrix composition is critical for the correct timing of both chondrocyte maturation and osteoblast differentiation and suggests that cartilage matrix proteoglycans control the diffusion of signaling factors that can both stimulate and repress these processes *in vivo*.

## NEW GENETICALLY ENCODED TRANSGENIC TOOLS RELEVANT TO SKELETAL RESEARCH

Calcium levels are tightly regulated in bone, and calcium transport and levels are critical for both osteoblast and osteoclast activity (Blair et al., 2011; Caudarella et al., 2011; Zhou et al., 2011). A variety of genetically encoded fluorescent biosensors have been developed over recent years, which undergo conformational changes and changes in fluorescent emission upon binding calcium, e.g., gCaMP (Muto and Kawakami, 2011; Muto et al., 2011). These could be fused to osteoblast or chondrocyte promoters to give a real-time read out of calcium signaling during skeletogenesis.

Another biologically encoded protein “MiniSOG,” the name is derived from Mini Singlet Oxygen Generator (Shu et al., 2011), could also prove useful as a tool for skeletal research. The MiniSOG protein is fluorescent and can therefore be tracked *in vivo*. However, in addition to its fluorescence it also acts, following photoconversion, as an oxygen generator that can generate electron dense substrates that are visible by electron microscopy. This approach has been used *in vivo*, in *C. elegans* successfully;** raising the prospect that the protein could be used in vertebrate species in some circumstances, for example in the fin of zebrafish which can easily be imaged, and are amenable to soaking in DAB and to photoactivation. This tool could potentially be used to tag and track growth factors in real time as they are produced, secreted, diffuse, and bind their targets fluorescently, then detected at an ultrastructural level by electron microscopy.

In summary, transgenic tools in mouse have been instrumental in uncovering how skeletogenesis and skeletal homeostasis are controlled *in* *vivo*. More recently, the development of relevant zebrafish and medaka transgenic lines have increased the utility of these models for skeletal research, complementing *in vitro* and mouse models. Moreover, these tools are increasingly allowing the dynamic observation of skeletal gene activation and signaling at a cellular level. The range of transgenic lines for skeletal research is likely to increase exponentially in the future as both the technology to generate them improves for all species and as the tools to manipulate, track, and study cell behavior and signaling continue to be developed, offering the tantalizing prospect of both visualizing and dissecting skeletal signaling as it occurs *in vivo*.

## Conflict of Interest Statement

The authors declare that the research was conducted in the absence of any commercial or financial relationships that could be construed as a potential conflict of interest.
